# Statistical Modeling of the Seismic Moments via Mathai Distribution

**DOI:** 10.3390/e24050695

**Published:** 2022-05-14

**Authors:** Pedro Vega-Jorquera, Erick De la Barra, Héctor Torres, Yerko Vásquez

**Affiliations:** 1 Departamento de Física, Facultad de Ciencias, Universidad de La Serena, La Serena 1720236, Chile; pvega@userena.cl (P.V.-J.); yvasquez@userena.cl (Y.V.); 2 Departamento de Matemáticas, Facultad de Ciencias, Universidad de La Serena, La Serena 1720236, Chile; htorres@userena.cl

**Keywords:** superstatistics, parametric statistics, seismic moments, completeness magnitude

## Abstract

Mathai’s pathway model is playing an increasingly prominent role in statistical distributions. As a generalization of a great variety of distributions, the pathway model allows the studying of several non-linear dynamics of complex systems. Here, we construct a model, called the Pareto–Mathai distribution, using the fact that the earthquakes’ magnitudes of full catalogues are well-modeled by a Mathai distribution. The Pareto–Mathai distribution is used to study artificially induced microseisms in the mining industry. The fitting of a distribution for entire range of magnitudes allow us to calculate the completeness magnitude ($M_{c}$). Mathematical properties of the new distribution are studied. In addition, applying this model to data recorded at a Chilean mine, the magnitude $M_{c}$ is estimated for several mine sectors and also the entire mine.

## 1. Introduction

Studying complex systems has been a recurring and growing scientific challenge. As these are non-equilibrium systems that exhibit complicated inhomogeneous spatio-temporal dynamics, several statistical models have been proposed. Searching for models that adequately describe physical systems in driven stationary states has promoted the development by Tsallis [1] of non-extensive statistical mechanics as a generalization of Boltzmann–Gibbs statistical mechanics. Note that physical systems in a driven stationary state are far from the equilibrium state and could be characterized by long-range interactions, metastability, multifractality, and others. A new generalization of this was proposed by Beck and Cohen [2] through the introduction of a more general framework called superstatistics, which includes Tsallis’ statistics. Based on the fact that different generalized forms of entropy have been proposed to obtain distributions of systems out of equilibrium, Mathai proposed the pathway model [3]. Mathai demonstrated that Tsallis’ statistics and superstatistics are particular cases of the pathway model. According to experimental results, he observed the existence of a variety of distributions, which can be grouped into two families. Based on these findings, a pathway parameter that allows passing from one functional form to another was introduced. Thus, by means of the maximization of a generalized entropy he obtained the pathway model given by

$$f\left(x\right)=cx^{\gamma -1}{\left(1-b\left(1-\alpha \right)x^{\delta }\right)}^{\frac{\eta }{1-\alpha }},\;x\geq 0$$

Depending on the values of parameters α, γ, δ>0, *b*, η and for x>0, it is possible to obtain a great variety of distributions as special cases; above *c* is a normalizing constant. For instance, when pathway parameter α=q>1 and γ=1, b=1, η=1 and δ=1, Tsallis’ statistics is obtained. For α=1, γ−1=3/4, b=1 and δ=1, we get the Maxwell–Boltzmann density. For α<1 and parameters γ=1, b=1, δ=1 and η=1, we obtain a *q*-binomial distribution. For α≠0, γ<1, η=0, and arbitrary values of *b* and δ, the Pareto distribution is obtained, (for more cases see [3,4,5]). Another aspect [6] is that for α<1, the Mathai model remains as a generalized type-1 beta model in the real case. For α>1, the model assumes the generalized type-2 beta model for real *x*. That is, there is a range of α pathway parameters that superstatistic considerations do not cover. The Pareto distribution obtained from the Mathai model results by assigning specific values to characteristic parameters of the pathway model, but the Pareto distribution is not obtained as a limit case of the Mathai law, as proposed here.

In this article, we propose a new Pareto–Mathai distribution, obtained by means of a transformation of the exponential distribution, whereby we pass from one kind of random variable to another. For instance, when the new distribution is applied to earthquakes, we pass from seismic magnitude to seismic moment [7]. In this context, crucial problems with the application of the Gutenberg–Richter (GR) law [8] are faced by superstatistics [9]: First, deviations of the GR model for the data of upper extreme seismic magnitudes register are successfully explained by Tsallis formalism (e.g., [1,2,10,11,12,13] and references therein). Second, the completeness magnitude ($M_{c}$), that is, the lowest magnitude at which one hundred percent of the events in a space-time volume are detected, must be determined in order to apply the GR law correctly. Here, we fit the Pareto–Mathai distribution for the whole range of magnitudes expressed as seismic moments and we also use it to estimate $M_{c}$ by an analytical method. Additionally, we can point out that this proposal allows adding a new distribution to the family of Pareto distributions (see e.g., [14,15,16]).

The purpose of this article is to construct the Pareto–Mathai distribution, to analyze its properties and to apply it to an experimental data of microearthquakes induced by mining. To this end, using data from a Chilean underground mine as a real system, we show that this new proposed distribution fits very well with the recorded data of the whole range of magnitudes. This allows us to analytically calculate the completeness magnitude.

The paper is organized as follows. In Section 2, the Pareto–Mathai distribution is constructed by applying a transformation to the exponential distribution and combining it with the Mathai distributions. The Pareto distribution is obtained as a limiting case, and a multivariate generalization of the Pareto–Mathai function is proposed. In Section 3, we investigate some properties of the Mathai and Pareto–Mathai distributions. The density function’s behavior for the new model is analyzed as a function of its parameters; additionally, we estimate the parameters of the Mathai and Pareto–Mathai distributions via Maximum Likelihood Estimation. In Section 4, the Pareto–Mathai distribution is applied to micro-earthquakes by using data recorded in a real system (a mine in Chile), and the completeness magnitude is calculated for its sectors and also the entire mine. The discussion and conclusions are the subject of Section 5.

## 2. Pareto–Mathai Distribution

In this section, we set up the Pareto–Mathai distribution by applying to the Mathai distribution the same transformation used to pass from the exponential distribution to the Pareto law. If F(x) is an exponential distribution function, that is, $F_{\hat{X}}\left(x\right)=1-e^{-\lambda x}$, the transformation G(x)=F∘ln(x) corresponds to a Pareto distribution with parameter λ and threshold $x_{m}=1$.

In [13] it was shown that the distribution

$$p\left(x\right)=\frac{\lambda^{3n/2}\Gamma \left(c+3n/2\right)}{\Gamma (3n/2)\Gamma (c)}{\left(x-x_{0}\right)}^{3n/2-1}{\left[1+\left(x-x_{0}\right)\lambda \right]}^{-c-3n/2}$$

for suitable parameters c,n,λ>0 and $x_{0}$ fits very well with the magnitudes of Chilean earthquakes. Taken n=2γ/3, λ=(q−1)b,c=−1/(1−q)−3n/2 and $x_{0}={\bar{x}}_{m}$ we obtain the Mathai distribution (shifted model)

(1) $$f\left(x\right)=c{\left(x-{\bar{x}}_{m}\right)}^{\gamma -1}{\left(1-\left(1-q\right)b\left(x-{\bar{x}}_{m}\right)\right)}^{\frac{1}{1-q}},\;x>{\bar{x}}_{m},$$

with q,b,γ>0 and $1-\left(1-q\right)b\left(x-{\bar{x}}_{m}\right)>0$ which modelizes the whole range of Chilean earthquakes magnitudes represented by a random variable $\hat{X}$. The distribution (1) can be obtained as a Beck Cohen superstatistics:

(2) $$f\left(x\right)=\int_{0}^{\infty }\rho \left(x\right)\beta e^{-\beta x}f_{\beta }\left(\beta \right)d\beta ,$$

where $\rho \left(x\right)={\left(x-{\bar{x}}_{m}\right)}^{\gamma -1}$ is a density of states and $f_{\beta }$ is the density function for the relevant parameter β that follows a chi-square distribution. In the Beck Cohen formalism, we say that f(x) results from considering the system locally explained by an exponential distribution.

We want to fit the empirical distribution of earthquake seismic moments represented by *X* because the seismic moment is fundamentally superior to any magnitude scale given the fact that it quantifies a parameter of the commonly accepted earthquake source model [17]. This construction is based on the facts that the Mathai distribution (1) fits magnitudes and seismic moments are usually modeled by a Pareto distribution. Therefore, we apply the transformation $F_{X}\left(x\right)=F_{\hat{X}}\left(\ln x\right)$, that is, $f_{X}\left(x\right)=\frac{1}{x}f_{\hat{X}}\left(\ln x\right)$, to obtain such a distribution. Hence, the seismic moments could be explained by a distribution

(3) $$f_{X}\left(x\right)=\frac{c}{x}{\left(\ln\frac{x}{x_{m}}\right)}^{\gamma -1}{\left(1-\left(1-q\right)b\ln\frac{x}{x_{m}}\right)}^{\frac{1}{1-q}},\;x>x_{m},$$

where $x_{m}=e^{{\bar{x}}_{m}}andc=c_{q,b,\gamma }$ is a normalization constant given by [3]:

(4) $$c_{q}=\left\{\begin{array}{cc} \frac{{\left[(q-1)b\right]}^{\gamma }}{B\left(\gamma ,\frac{1}{q-1}-\gamma \right)}, & q>1\\ \frac{{\left[(1-q)b\right]}^{\gamma }}{B\left(\gamma ,1+\frac{1}{1-q}\right)}, & q<1 \end{array}\right..$$

In this way, the distribution function for q>1 can be calculated compactly in terms of beta functions, namely

(5) $$\begin{array}{c} F\left(x\right)=I\left(\frac{s(x)}{s(x)+1};\gamma ,\frac{1}{q-1}-\gamma \right), \end{array}$$

where $s\left(x\right)=\left(q-1\right)b\ln\frac{x}{x_{m}}$ and I is given by

$$I\left(x;a,b\right)=\frac{B(x;a,b)}{B(a,b)},$$

where B is the incomplete beta function:

$$B\left(x;a,b\right)=\int_{0}^{x}u^{a-1}{\left(1-u\right)}^{b-1}du,\;0\leq x\leq 1$$

On other hand, for q<1, the cumulative distribution function is given by

$$F\left(x\right)=I\left(\frac{r(x)}{r(x)+1};\gamma ,1+\frac{1}{1-q}\right),$$

where $r\left(x\right)=\left(1-q\right)b\ln\frac{x}{x_{m}}$. The expression for the distribution function is specially useful for applications because it allows us to compute the survival function.

**Remark** **1.** *An important limit case of the Pareto–Mathai law (3) is when q→1, which yields the shifted log-gamma distribution [18]:*

(6) $$f\left(x\right)=c{\left(\ln\frac{x}{x_{m}}\right)}^{\gamma -1}\frac{x_{m}^{b}}{x^{b+1}},\;x>x_{m},$$

*where c is the normalization constant $c=b^{\gamma }/\Gamma \left(\gamma \right)$ (here Γ(.) is the gamma function).*

**Remark** **2.** *Another way to obtain the Pareto–Mathai distribution (3) is via the Beck–Cohen superstatistics:*

$$f\left(x\right)=\int_{0}^{\infty }\rho \left(x\right)\beta \frac{x_{m}^{\beta }}{x^{\beta +1}}f_{\beta }\left(\beta \right)d\beta ,$$

*where the density $f_{\beta }$ is the same as in (2) (that is, a chi-square distribution) and the density of states is $\rho \left(x\right)=\ln^{\gamma -1}\left(x/x_{m}\right)$.*

**Remark** **3.** *Inspired by the more general form of the Mathai distribution, the density (3) could be generalized as follows:*

(7) $$f\left(x\right)=\frac{c}{x}\ln^{\gamma -1}\frac{x}{x_{m}}{\left(1-\left(1-q\right)b\ln^{\delta }\frac{x}{x_{m}}\right)}^{\frac{\eta }{1-q}},\;x\geq x_{m},$$

*where $c=c_{q}$ is given by*

(8) $$c_{q}=\left\{\begin{array}{cc} \frac{\delta {\left[(q-1)b\right]}^{\frac{\gamma }{\delta }}}{B\left(\frac{\gamma }{\delta },\frac{\eta }{q-1}-\frac{\gamma }{\delta }\right)}, & q>1\\ \frac{\delta {\left[(1-q)b\right]}^{\frac{\gamma }{\delta }}}{B\left(\frac{\gamma }{\delta },1+\frac{\eta }{1-q}\right)}, & q<1 \end{array}\right.,$$

*where $\frac{\eta }{q-1}>\frac{\gamma }{\delta }>0$. It is easy to verify that the constant c is the same as for the Mathai density*

(9) $$f\left(x\right)=c{\left(x-x_{m}\right)}^{\gamma -1}{\left(1-\left(1-q\right)b{\left(x-x_{m}\right)}^{\delta }\right)}^{\frac{\eta }{1-q}},\;x>x_{m}.$$

*Additionally, we can show that the distribution function given by*

(10) $$F\left(x\right)=\left\{\begin{array}{cc} I\left(\frac{s(x)}{s(x)+1};\frac{\gamma }{\delta },\frac{\eta }{q-1}-\frac{\gamma }{\delta }\right), & q>1,\\ I\left(\frac{r(x)}{r(x)+1};\frac{\gamma }{\delta },1+\frac{\eta }{1-q}\right), & q<1, \end{array}\right.$$

*corresponds to (7) for $s\left(x\right)=\left(q-1\right)b\ln^{\delta }\frac{x}{x_{m}}$ and $r\left(x\right)=\left(1-q\right)b\ln^{\delta }\frac{x}{x_{m}}$, and to (9) for $s\left(x\right)=\left(q-1\right)b{\left(x-x_{m}\right)}^{\delta }$ and $r\left(x\right)=\left(1-q\right)b{\left(x-x_{m}\right)}^{\delta }$.*

## 3. Properties of Pareto–Mathai Distribution

An important difference between the Tsallis and Mathai distributions (9) is that the density function for the former is always strictly decreasing but for the latter it could have a maximum attained in a value greater than $x_{m}$ (depending on the parameter values).

**Proposition** **1.** *The density function (9) has a maximum at*

(11) $$x^{*}={\left(\frac{\gamma -1}{b[(\gamma -1)(1-q)+\delta \eta ]}\right)}^{1/\delta }+x_{m},$$

if (γ−1)(1−q)+δη>0, b>0, γ,q>1 and δ≥1. Moreover, (9) is monotonically increasing to the left of $x^{*}=x_{b,q,\gamma ,\delta ,\eta }^{*}$ and is monotonically decreasing to its right. Thus, under this condition, its distribution function has an inflection point at $x^{*}$.

**Proof.**  The derivative of f(x) is:

$$f^{\prime }\left(x\right)={\left(x-x_{m}\right)}^{\gamma -2}{\left(1-\left(1-q\right)b{\left(x-x_{m}\right)}^{\delta }\right)}^{\frac{\eta }{1-q}-1}\left\{\gamma -1-{\left(x-x_{m}\right)}^{\delta }\left[\left(\gamma -1\right)\left(1-q\right)b+\delta \eta b\right]\right\}.$$

Thus the critical values for f(x) are $x_{1}=x_{m}$ and $x_{2}={\left(\frac{\gamma -1}{b[(\gamma -1)(1-q)+\delta \eta ]}\right)}^{1/\delta }+x_{m}\geq x_{m}$. It is easily shown that $f^{\prime }\left(x\right)>0$$\forall x\in [x_{m},x_{2})$ and $f^{\prime }\left(x\right)<0$$\forall x\in (x_{2},\infty )$. On other hand, we have $f(x_{m})=0$ and f(x)≥0 then f(x) is the minimum at $x_{m}$ and the absolute maximum point will be $x={\left(\frac{\gamma -1}{b[(\gamma -1)(1-q)+\delta \eta ]}\right)}^{1/\delta }+x_{m}\geq x_{m}$. □

Similarly, the Pareto–Mathai’s density function (7) has an absolute maximum value. However, the critical value, where the maximum is reached, cannot be calculated in a closed form. Thus, numerical methods are necessary to compute it:

**Proposition** **2.** The density function (7) has a maximum at a point $x^{*}>x_{m}$ determined uniquely by the equation:

(12) $$\left(1-q\right)bu^{\delta +1}-\left[\left(\gamma -1\right)\left(1-q\right)+\eta \delta \right]bu^{\delta }-u+\gamma -1=0,\;u\geq 0$$

where $u=\ln\frac{x^{*}}{x_{m}}$, (γ−1)(1−q)+δη>0, b>0, γ,q>1, and δ≥1. Moreover, (7) is monotonically increasing to the left of $x^{*}$ and monotonically decreasing to its right. Thus, under this condition its distribution function has an inflection point at $x^{*}$.

*Further, the solution of (12) satisfies:*

(13) $$\begin{array}{cc} \; & \frac{\partial u}{\partial \gamma }=\frac{\left(q-1\right)bu^{\delta }+1}{\left(q-1\right)b\left(\delta +1\right)u^{\delta }+\left[\left(\gamma -1\right)\left(1-q\right)+\eta \delta \right]b\delta u^{\delta -1}+1}>0 \end{array}$$

(14) $$\begin{array}{cc} \; & \frac{\partial u}{\partial b}=-\frac{\left(q-1)u+[(\gamma -1)(1-q)+\eta \delta \right]}{\left(q-1\right)b\left(\delta +1\right)u^{\delta }+\left[\left(\gamma -1\right)\left(1-q\right)+\eta \delta \right]b\delta u^{\delta -1}+1}u^{\delta }<0. \end{array}$$

Thus, the extreme value $x^{*}$ is increasing in γ and is decreasing in b. Furthermore, if u<γ−1 we have:

(15) $$\frac{\partial u}{\partial q}=\frac{\left(-u+\gamma -1\right)}{\left(q-1\right)b\left(\delta +1\right)u^{\delta }+\left[\left(\gamma -1\right)\left(1-q\right)+\eta \delta \right]b\delta u^{\delta -1}+1}bu^{\delta }>0,$$

as $x^{*}$ is increasing in q. Additionally, the explicit expression of $x^{*}$ for the particular case δ=η=1, that is, for the distribution (1), is $x^{*}=x_{m}e^{u^{*}}$, where

(16) $$u^{*}=\frac{\left(\gamma -1\right)\left(1-q\right)b+b+1+\sqrt{{\left[(\gamma -1)(1-q)b+b+1\right]}^{2}+4\left(q-1\right)b\left(\gamma -1\right)}}{2b(q-1)}$$

**Proof.**  The equation $f^{\prime }\left(x\right)=0$, where f(x) given by (7), is equivalent to:

$$\begin{array}{cc} \frac{1}{x^{2}}\ln^{\gamma -2}\frac{x}{x_{m}} & {\left(1-\left(1-q\right)b\ln^{\delta }\frac{x}{x_{m}}\right)}^{\frac{\eta }{1-q}-1}\times \\ \; & \times \left[\left(1-q\right)b\ln^{\delta +1}\frac{x}{x_{m}}-\left[\left(\gamma -1\right)\left(1-q\right)+\eta \delta \right]b\ln^{\delta }\frac{x}{x_{m}}-\ln\frac{x}{x_{m}}+\gamma -1\right]=0. \end{array}$$

Thus, the critical points are given by

$$\begin{array}{c} x_{1}=x_{m},\\ \left(1-q\right)bu^{\delta +1}-\left[\left(\gamma -1\right)\left(1-q\right)+\eta \delta \right]bu^{\delta }-u+\gamma -1=0, \end{array}$$

where $u=\ln\frac{x_{2}}{x}$. At $x_{1}=x_{m}$ we have $f(x_{1})=0$ and f(x)≥0 for all $x>x_{m}$ hence $x_{1}=x_{m}$ is a global minimum of (3). Now, we are going to prove that the density is maximum at $x_{2}$, which is uniquely determined by (12). Define

(17) $$g\left(u\right)=\left(1-q\right)bu^{\delta +1}-\left[\left(\gamma -1\right)\left(1-q\right)+\eta \delta \right]bu^{\delta }-u+\gamma -1.$$

Note that g(0)=γ−1>0 by assumption and $g(u_{M})<0$ for $u_{M}$ is big enough because the coefficient of the term $u^{\delta +1}$ is negative. Thus, by the Intermediate Value Theorem there is a solution for (12). On the other hand $g^{\prime }\left(u\right)<0$ for all u>0 given that (γ−1)(1−q)+ηδ>0. Clearly $f^{\prime }\left(x\right)>0$ for $\left[x_{m},x_{max}\right)$ and $f^{\prime }\left(x\right)<0$ for $\left(x_{max},\infty \right)$. Finally, the relations (13)–(15) are obtained by implicitly deriving the Equation (12). These analytical results are obtained by varying the probability density function f(x) as a function of the seismic moments for different values of the parameters *q*, γ and *b*. Accordingly, to study the behavior of f(x) with respect to one of them, the others must be considered constant. □

**Remark** **4.** *The Equations (11) and (16) give us, respectively, the completeness magnitude using the Mathai distribution and the completeness seismic moment using the Pareto–Mathai distribution.*

**Figure 1:**
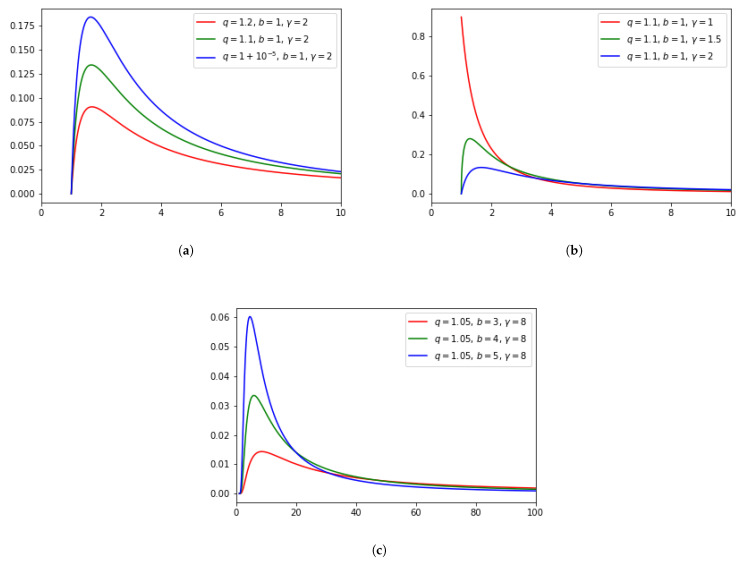
Pareto–Mathai density function f(x) for different values of the parameters *q*, *b* and γ. In (**a**), *b* and γ are fixed. In (**b**), *b* is fixed. In (**c**) *q* is fixed.

The Pareto–Mathai density function (3) is represented in Figure 1 for different values of the parameters *q*, *b* and γ. Figure 1a shows that the density function begins with a minimum value, increases to a maximum value and subsequently decreases. Assuming fixed values of the parameters *b* and γ, the maximum value of the density function increases when the entropic index *q* decreases, approaching value one. Thus, the effect of increasing the value of *q* implies an increase in the value of the density function for the high values of the random variable *x*, thus generating a heavy-tailed distribution. In Figure 1b, we assume that the parameters *q* and *b* are fixed and let the γ parameter vary. We observe that when γ decreases, the maximum of the density function increases and, depending on the chosen values of *q* and *b*, its position shifts towards the lower *x* values. In the limit case γ=1, the density function is a strictly decreasing function. This limit case corresponds to the Beck–Cohen superstatistical model in which the density of states is one, i.e., ρ(x)=1. In the region of lower values of the random variables, the behavior of the density function is clearly controlled by the density of states. In Figure 1c, we consider the entropic parameters *q* and γ fixed and let the values of the parameter *b* vary. When the value of *b* increases, the maximum value increases and the position of the maximum shifts towards the lower values of *x*. The density function increases from a minimum up to a maximum value and subsequently decreases. However, in the region of large values of the random variable, the density function does not vary significantly as a function of the parameter *b*.

A comparison between the Pareto distribution and the Pareto–Mathai distribution demonstrates that moments for Pareto exist for the shape parameter greater than one, while for Pareto–Mathai, no moment exists at all. We summarize these observations in the following two propositions. The first is about Mathai distribution [19] and the second is about the Pareto–Mathai law.

**Proposition** **3.** *For the Mathai distribution (9) with $x_{m}=0$, the j-th moment for q>1 is given by*

$$E\left(X^{j}\right)=\frac{c_{q}}{\delta {\left(b\left(q-1\right)\right)}^{\frac{\gamma +j}{\delta }}}B\left(\frac{\gamma +j}{\delta },\frac{\eta }{q-1}-\frac{\gamma +j}{\delta }\right),$$

*where $c_{q}$ is given by (8), $\frac{\eta }{q-1}-\frac{\gamma }{\delta }>0$, $\frac{\eta }{q-1}-\frac{\gamma +j}{\delta }>0$, γ+m>0 and γ,b,η,δ>0. For q<1 the j-th moment is given by*

$$E\left(X^{j}\right)=\frac{c_{q}}{\delta {\left(b\left(1-q\right)\right)}^{\frac{\gamma +j}{\delta }}}B\left(\frac{\gamma +j}{\delta },1+\frac{\eta }{1-q}\right),$$

*where $c_{q}$ is given by (8), γ+j>1, γ,δ,b>0.*

**Remark** **5.** *For the shifted Mathai distribution (9) we have by direct calculus:*

E(Xj)=∑i=0jjixmj−iE(X0i),

*where $E(X_{0}^{0})=1$ and $E(X_{0}^{i})$ is the i-th moment of the unshifted Mathai distribution given by Proposition 3 for i=0,1,…,j.*

**Proposition** **4.** *Let η,b>0, δ≥1 and γ>1. For the Pareto–Mathai distribution (7) and for q>1, there does not exist any moment. Instead, for 0<q<1, the j-th moment exists. In particular, the expectation is given by*

$$E\left(X\right)=\frac{c_{q}}{\delta {\left[\left(1-q\right)b\right]}^{\frac{\gamma }{\delta }}}B\left(1+\frac{\eta }{1-q},\frac{\gamma }{\delta }\right)$$

**Proof.**  Suppose q>1. Then

$$\begin{array}{cc} E(X) & =c\int_{x_{m}}^{\infty }\ln^{\gamma -1}\frac{x}{x_{m}}{\left(1-\left(1-q\right)b\ln^{\delta }\frac{x}{x_{m}}\right)}^{\frac{\eta }{1-q}}dx\\ \; & =\frac{cx_{m}}{\delta {\left[\left(q-1\right)b\right]}^{\frac{\gamma }{\delta }}}\int_{1}^{\infty }u^{\frac{\gamma }{\delta }-1}{\left(1+u\right)}^{\frac{\eta }{1-q}}e^{\frac{u^{1/\delta }}{{\left[\left(q-1\right)b\right]}^{1/\delta }}}du \end{array}$$

The integrand of the last integral is continuous and non negative and goes to infinity if u→∞, whence this integral diverges.

Now, suppose q<1. The support of f(x) is $\left(x_{m},x_{m}e^{{\left[\left(1-q\right)b\right]}^{-1/\delta }}\right)$. Clearly, the integrand of $E(X^{j})$, $g\left(x\right)=cx^{j-1}\ln^{\gamma -1}\left(x/x_{m}\right){\left(1-\left(1-q\right)b\ln^{\delta }\left(x/x_{m}\right)\right)}^{\eta /(1-q)}$ is continuous function over the compact interval $\left[x_{m},x_{m}e^{{\left[\left(1-q\right)b\right]}^{-1/\delta }}\right]$ so $E(X^{j})$ is finite. For j=1 we have:

$$E\left(X\right)=\frac{c}{\delta {\left[\left(1-q\right)b\right]}^{\frac{\gamma }{\delta }}}\int_{0}^{1}u^{\frac{\gamma }{\delta }-1}{\left(1-u\right)}^{\frac{\eta }{1-q}}du=\frac{c_{q}}{\delta {\left[\left(1-q\right)b\right]}^{\frac{\gamma }{\delta }}}B\left(1+\frac{\eta }{1-q},\frac{\gamma }{\delta }\right).$$

□

### 3.1. Multivariate Pareto–Mathai Distribution

The Pareto–Mathai distribution can be generalized to the multivariate case, just as Mathai’s model was generalized by Joseph [19]:

$$\begin{array}{cc} \; & f\left(x_{1},x_{2},\ldots ,x_{n}\right)=\frac{k_{q}}{x_{1}\cdot x_{2}\ldots \cdot x_{n}}{\left(\ln\frac{x_{1}}{x_{m}^{1}}\right)}^{\gamma_{1}-1}{\left(\ln\frac{x_{2}}{x_{m}^{2}}\right)}^{\gamma_{2}-1}\ldots {\left(\ln\frac{x_{n}}{x_{m}^{n}}\right)}^{\gamma_{n}-1}\times \\ \; & \times {\left(1-\left(1-q\right)\left[b_{1}\ln^{\delta_{1}}\frac{x_{1}}{x_{m}^{1}}+b_{2}\ln^{\delta_{2}}\frac{x_{2}}{x_{m}^{2}}+\ldots +b_{n}\ln^{\delta_{n}}\frac{x_{n}}{x_{m}^{n}}\right]\right)}^{\frac{\eta }{1-q}},\;x_{i}>x_{m}^{i}\;\left(i=1,2,\ldots ,n\right) \end{array}$$

where

(18) $$k_{q}=\frac{\prod_{i=1}^{n}\delta_{i}{\left(b_{i}\left(q-1\right)\right)}^{\frac{\gamma_{i}}{\delta_{i}}}}{B\left(\frac{\eta }{q-1}-\sum_{i=1}^{n}\frac{\gamma_{i}}{\delta_{i}},\sum_{i=1}^{n}\frac{\gamma_{i}}{\delta_{i}}\right)},\;q>1$$

and

(19) $$k_{q}=\frac{\prod_{i=1}^{n}\delta_{i}{\left(b_{i}\left(q-1\right)\right)}^{\frac{\gamma_{i}}{\delta_{i}}}}{B\left(1+\frac{\eta }{1-q},\sum_{i=1}^{n}\frac{\gamma_{i}}{\delta_{i}}\right)},\;q<1.$$

Above $x_{m}^{i},\delta_{i},\gamma_{i}\geq 0$, for i=1,…,n and $\frac{\eta }{q-1}-\sum_{i=1}^{n}\frac{\gamma_{i}}{\delta_{i}}>0$ if q>1 and $1+\frac{\eta }{1-q}>0$ if q<1.

The multivariate models whose marginal distribution are Pareto–Mathai and Mathai distributions are important in seismology, since the inter-event time can be modeled by a *q*-exponential distribution and the seismic moments can be modeled by a Pareto–Mathai law. In particular, the multivariate Pareto-*q*-Exponential-Mathai distribution can be defined as:

$$\begin{array}{cc} \; & f\left(x_{1},\ldots ,x_{l},x_{l+1},\ldots ,x_{n}\right)=k_{q}\frac{{\left(x_{1}-x_{m}^{1}\right)}^{\gamma_{1}-1}\cdot \ldots \cdot {\left(x_{l}-x_{m}^{l}\right)}^{\gamma_{l}-1}}{x_{l+1}\cdot \ldots \cdot x_{n}}\ln^{\gamma_{l+1}-1}\frac{x_{l+1}}{x_{m}^{l+1}}\ldots \ln^{\gamma_{n}-1}\frac{x_{n}}{x_{m}^{n}}\times \\ \; & \times {\left(1-\left(1-q\right)\left[b_{1}{\left(x_{1}-x_{m}^{1}\right)}^{\delta_{1}}+\ldots +b_{l}{\left(x_{l}-x_{m}^{l}\right)}^{\delta_{l}}+b_{l+1}\ln^{\delta_{l+1}}\frac{x_{l+1}}{x_{m}^{l+1}}+\ldots +b_{n}\ln^{\delta_{n}}\frac{x_{n}}{x_{m}^{n}}\right]\right)}^{\frac{\eta }{1-q}}, \end{array}$$

where $x_{i}>x_{m}^{i}$ for all i=1,2,…,n, $k_{q}$ is the normalization constant given by (18) and (19), $x_{m}^{i},\delta_{i},\gamma_{i}\geq 0$ for i=1,…,n, $\frac{\eta }{q-1}-\sum_{i=1}^{n}\frac{\gamma_{i}}{\delta_{i}}>0$ if q>1 and $1+\frac{\eta }{1-q}>0$ if q<1. Here, the first *l* variables are Mathai distributed and the remaining n−l variables are Pareto–Mathai distributed.

### 3.2. Parameter Estimation

To estimate the parameters for a dataset, we write the optimization problems to find the optimal parameters for Mathai and Pareto–Mathai distributions. As we can observe, the optimality conditions for the optimal parameters are rather complicated, so numerical methods for non linear optimization problems must be used in order to find these parameters. The optimization problems was implemented in Python and the scipy.optimize package with the Broyden-Fletcher-Goldfarb-Shanno algorithm was used. A logarithmic transformation was applied to the data and Mathai distribution parameters was estimated by MLE in order to provide a good initial guess for the parameters in the Pareto Mathai distribution. The logarithmic transformation was used because the optimization problem for transformed data is more stable than the problem for the original data.

Let $\left(x_{1},x_{2},\ldots ,x_{N}\right)$ be a simple sample. Then, the likelihood function for (7) is given by:

$$L=L\left(q,b,\gamma ,\delta ,\eta \right)=\prod_{i=1}^{N}f\left(x_{i};q,b,\gamma ,\delta ,\eta \right)=\prod_{i=1}^{N}\frac{c_{q,b,\gamma }}{x_{i}}{\left(\ln\frac{x_{i}}{x_{m}}\right)}^{\gamma -1}{\left(1-\left(1-q\right)b\ln^{\delta }\frac{x_{i}}{x_{m}}\right)}^{\frac{\eta }{1-q}}.$$

Note that the parameter $x_{m}$ is not estimated using the Maximum Likelihood Estimation. It is obtained directly from the data as the minimum seismic moment.

In order to facilitate the optimization of L(q,b,γ,δ,η), we take the logarithm:

$$\ln L=N\ln c_{q,b,\gamma }-\sum_{i=1}^{N}\ln x_{i}+\left(\gamma -1\right)\sum_{i=1}^{N}\ln\left(\ln\frac{x_{i}}{x_{m}}\right)+\frac{\eta }{1-q}\ln\left(1-\left(1-q\right)b\ln^{\delta }\frac{x_{i}}{x_{m}}\right).$$

Thus the maximization problem $\max_{q,b,\gamma ,\delta ,\eta }L\left(q,b,\gamma ,\delta \right)$ is equivalent to

$$\begin{array}{cc} \max_{q,b,\gamma ,\delta ,\eta } & \left[N\ln\frac{{\left[(q-1)b\right]}^{\gamma }}{B\left(\frac{\gamma }{\delta },\frac{\eta }{q-1}-\frac{\gamma }{\delta }\right)}+\left(\gamma -1\right)\sum_{i=1}^{N}\ln\left(\ln\frac{x_{i}}{x_{m}}\right)+\frac{\eta }{1-q}\ln\left(1-\left(1-q\right)b\ln^{\delta }\frac{x_{i}}{x_{m}}\right)\right]\\ \mathrm{such}\;\mathrm{that} & \;\frac{\eta }{q-1}-\frac{\gamma }{\delta }\geq 0\\ \; & \;q,b,\gamma ,\delta ,\eta \geq 0. \end{array}$$

**Remark** **6.** *The maximization of the likelihood function for (9) is equivalent to the following optimization problem:*

$$\begin{array}{cc} \max_{q,b,\gamma ,\delta ,\eta } & \left[N\ln\frac{{\left[(q-1)b\right]}^{\gamma }}{B\left(\gamma ,\frac{\eta }{q-1}-\frac{\gamma }{\delta }\right)}+\left(\gamma -1\right)\left(\sum_{i=1}^{N}\ln\left(x_{i}-x_{m}\right)\right)+\frac{\eta }{1-q}\ln\left(1-\left(1-q\right)b{\left(x_{i}-x_{m}\right)}^{\delta }\right)\right]\\ such\;that & \;\frac{\eta }{q-1}-\frac{\gamma }{\delta }\geq 0\\ \; & \;q,b,\gamma ,\delta ,\eta \geq 0. \end{array}$$

By solving this optimization problem numerically, we will give the estimate of the parameters of the Pareto–Mathai model. For optimizing, we use an optimization python package.

## 4. Application

**Figure 2:**
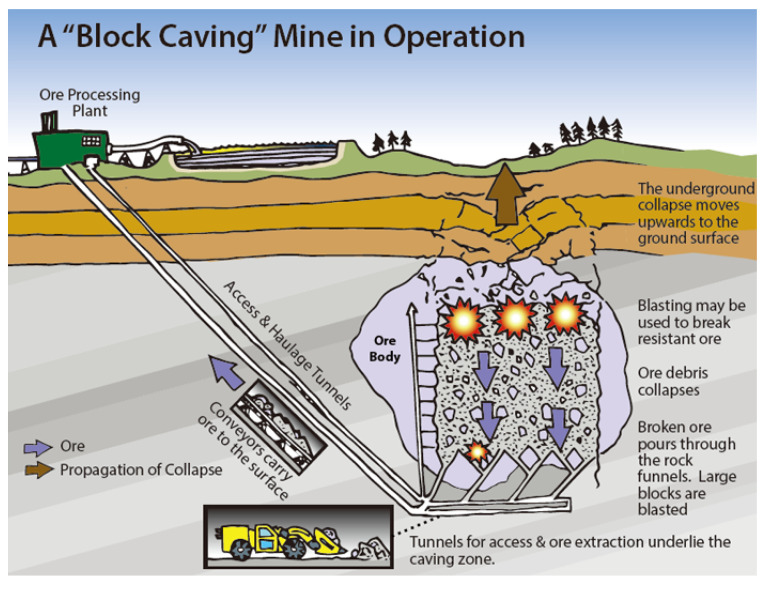
Block caving scheme http://www.groundtruthtrekking.org/Graphics/block-caving-underground-mining-method-diagram.html (accessed on 5 September 2021).

Block/panel caving is a common technique to mine typically low-grade massive steeply dipping ore bodies with high friability (see Figure 2). An undercut with haulage access is driven under the orebody, with “drawbells” being excavated between the top of the haulage level and the bottom of the undercut. The orebody is drilled and blasted above the undercut to create a void at each draw-point, so that the rock breaks and falls due to friability and gravity. After that, the broken ore is removed via the haulage access.

Rock removal generates instability in the ore body and new fracturing begins. This process produces seismic activity, which we examine in this section using the Pareto–Mathai distribution (1). The seismic moments can be modeled with Pareto distribution and its variations such as the *q* − Pareto and the Pareto–Mathai distribution. Additionally, the seismic moment scale is more accurate than magnitude scale because the magnitude generally is expressed at most with two decimals, implying a significant loss of information on energy [12].

**Figure 3:**
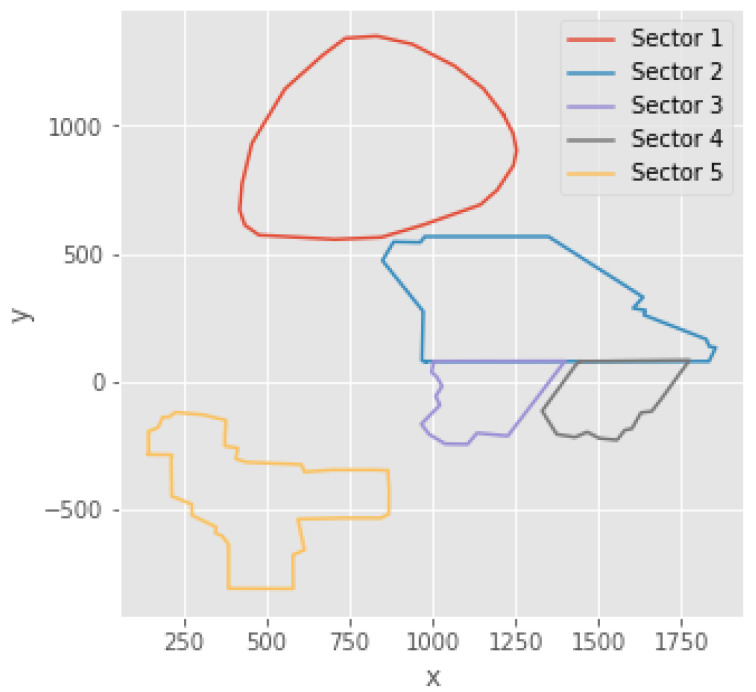
Graphic representation of the mine sectors. They are considered as one system.

An underground mine comprises a set of sectors, which are exploited independently. However, these sectors are interrelated because the rock extracted is transported at a transportation level, which is the same for all sectors. Additionally, the sectors have shared geophysical aspects. For example, seismic activity in one sector could influence seismicity in another. In our study case, we consider a mine represented in Figure 3, which is a Chilean underground mine divided into five sectors, covering approximately 2000×2500 m^2^. The first four sectors are close to each other and the fifth is farther away from the previous ones.

The data is expressed by the Moment Magnitude Scale ($M_{0}$) for magnitudes and in Newton-meters (N·m) for seismic moments. The relation between magnitudes and seismic moments is given by [7]:

$$M_{w}=\frac{3}{2}\left(\log_{10}M_{0}-9.1\right),$$

where $M_{w}$ is the moment magnitude scale or simply the magnitude. Note that $M_{0}$ is measured in energy units, while the magnitude $M_{w}$ is an dimensionless quantity which can be negative. In mining, many tremors with negative magnitudes are detected by geophones, which are low energy events.

The seismic moment data obtained from this mine considers events from all spectre of magnitudes, including even unreliable data. The limitation of the instruments (geophones) to measure magnitude earthquakes produces unreliable measures under a magnitude known as a completeness magnitude ($M_{c}$). This happens because small tremors are detected only if they occur sufficiently close to a geophone. Thus, the number of these tremors are underestimated. The estimation of the magnitude $M_{c}$ is fundamental to distinguish the reliable data from the unreliable and to do so we will fit the Pareto–Mathai distribution and calculate its critical value via Equation (16).

The database used includes events from 2003 to 2007 and the mine is partitioned into five sectors, associating a sub-database with each one as follows: sector 1:71,621 data; sector 2:70,987 data; sector 3:3641 data; sector 4:24,770 data and sector 5:8996 data. Later, a complete database for the entire mine was considered. The range of values of the seismic magnitudes and their corresponding seismic moments are indicated in Table 1.

### 4.1. Cumulative Probability

**Figure 4:**
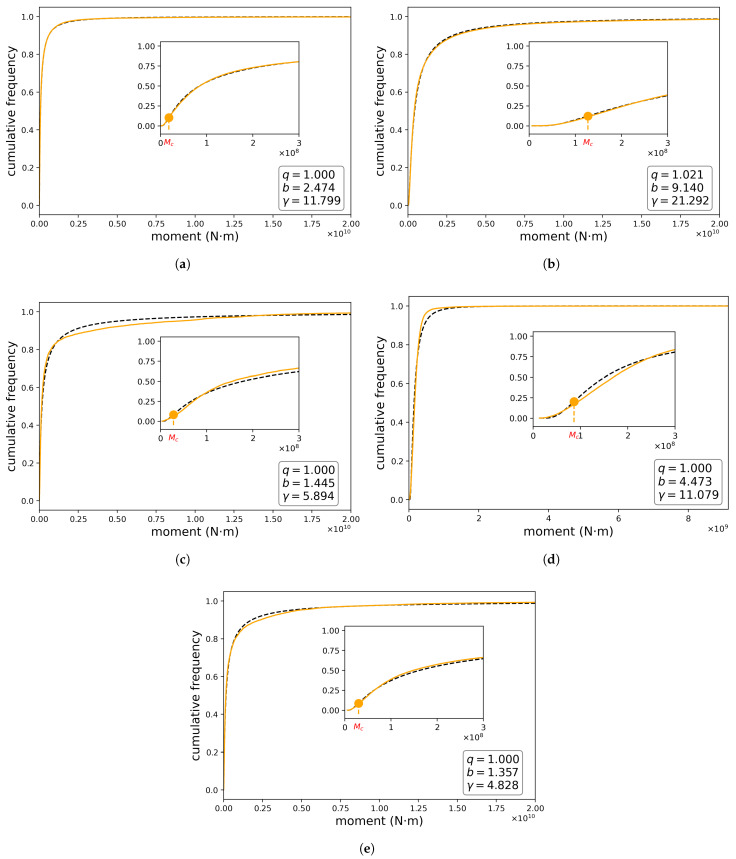
Frequency distributions for seismic moments at different mine sectors. The empirical distribution appears in orange and the black dashed line is the theoretical distribution.

**Figure 5:**
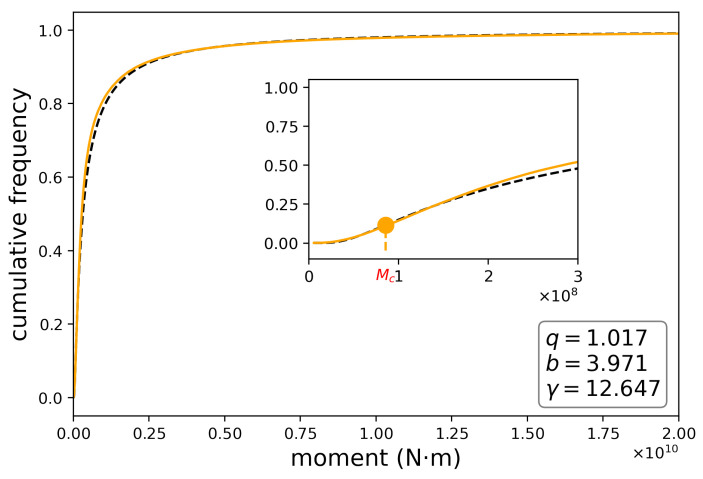
Frequency distribution for a set of sectors. The empirical distribution appears in orange and the black dashed line is the theoretical distribution.

By using our proposed model, we fit the cumulative probability, corresponding to Equation (5), as a function of seismic moments. Figure 4 reveals a very good fit between the theoretical model and the data recorded in each sector. This can be observed by means of the RMSE, $R^{2}$ and MAPE values (Table 2). In sectors 2 and 3, the *q* values are greater than one. However, the γ values vary widely and are significantly greater than one: in sector 1, Figure 4a, γ=11.778; in sector 2, Figure 4b, γ=14.999; in sector 3, Figure 4c, γ=3.754; in sector 4, Figure 4d, γ=11.242 and in sector 5, Figure 4e, γ=4.827. This clearly shows that the term representing the “density of states” is very different from one and differ among some sectors, which implies different completeness magnitudes. If we consider the mine as a total system made up of five sectors, we find that the cumulative distribution that best fits the recorded data is the one whose parameters are: q=1.017; b=3.966 and γ=12.641, (Figure 5). The result demonstrates that q>1 for Sectors 2 and 3, whence the data presents a deviation from the log-gamma distribution; in Sectors 1,3 and 4, the opposite happens. Since Sector 1 presents the biggest number of events among all sectors and the data in this sector behaves according to a log-gamma distribution, if we consider the data of the whole mine it is modeled by a log-gamma distribution as well (see Figure 5). On the other hand, in the region with the lowest values of the seismic moments, the system behaves according to the Pareto–Mathai distribution that we are proposing and which incorporates a “state density” term different from unity. The cumulative distribution function, F(x), starts from a minimum value at $x_{m}$ and then increases reaching an inflection point where it changes from a positive to a negative concavity. This inflection point is the completeness magnitude. Equivalently, the probability density f(x), which starts from a minimum value, then increases up to a maximum value and finally decreases.

In the next section, we analyze the density function of the seismic moments according to the behavior of the cumulative distribution function. The inflection point is expected to behave according to the interplay of parameters *q*, *b* and γ. Importantly, for lower values of the parameter γ, the effect of the density of states is lower and the behavior of the cumulative distribution function will depend fundamentally on the parameters *q* and *b*. Thus, in the limit γ→1, our model tends to the *q*–Pareto model [20] or the Beck–Cohen model with state density ρ(x)=1.

### 4.2. Density Probability

The behavior of the density of probability function (PDF) is analyzed regarding its dependence on the parameters *b*, *q* and γ. This theoretical model includes studying the variation of the PDF on one parameter while keeping the others constant. In this context, and considering Equations (14) and (15), we have the following cases: an increment (decrement) in entropic parameter *q* implies an increment (decrement) in $M_{c}$. Secondly, for parameter γ, we have a similar situation as for *q*. Thirdly, for the *b* parameter we have the inverse situation: an increment (decrement) in *b* implies a decrement (increment) in $M_{c}$. The completeness magnitudes for each sector are shown in Table 3.

**Figure 6:**
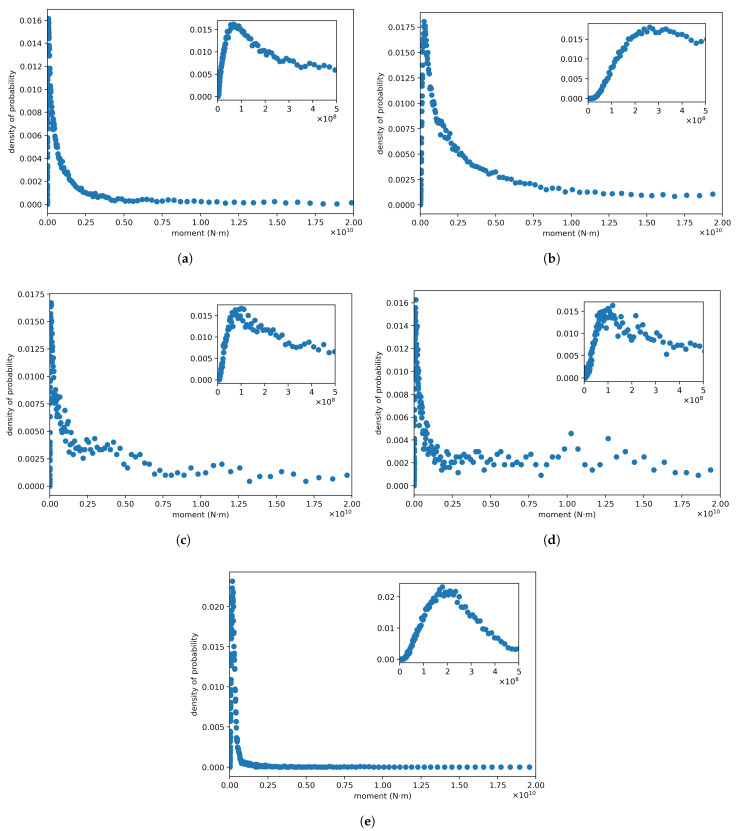
Densities for seismic moments at different mine sectors. The figures (**a**–**d**) corresponds to densities for sectors 1–4 respectively. The figure (**e**) is the probability density function for the seismic moments registered in the whole mine.

In order to study the behavior of the density function as a function of the indicated parameters using data recorded at a mine, in Figure 6, we represent the probability density of the seismic moment. The data are the same as in Figure 4. The behavior of the probability density function in the region of lower values of seismic moments is similar to that expected under the Pareto–Mathai model that we propose (Proposition 2). The probability density starts with a minimum value, increases up to a maximum at $M_{c}$ and subsequently decreases with the increase in the value of the seismic moments. The behavior of the probability density function f(x) as a function of parameters *q*, γ and *b* depends on the interplay between these parameters as we mentioned in Section 3.

**Figure 7:**
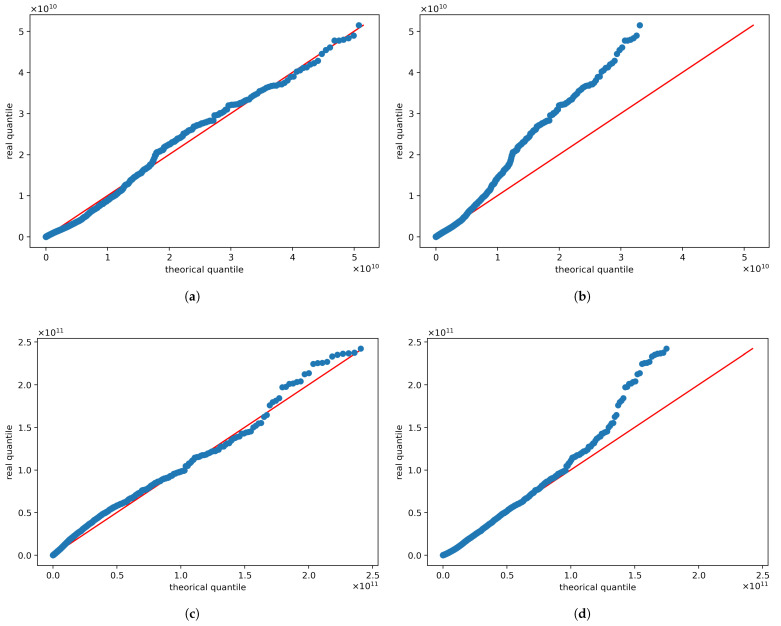
QQ-plots for Moment Distributions. In the first column are shown the QQ-plot for Pareto–Mathai distribution. More specifically the figures (**a**,**c**) correspond to the Pareto–Mathai QQ-plot for sectors 1 and 2 respectively. In the second column are shown the QQ-plot for log-gamma distribution (the limit case of Pareto–Mathai law). The figures (**b**,**d**) are the log-gamma QQ-plots for sectors 1 and 2 respectively.

Our Pareto–Mathai model fits very well the empirical distribution of the seismic moments for most sectors, encompassing values corresponding to the whole range of detected magnitudes. This allows us to estimate analytically the completeness magnitude $M_{c}$, as can be observed by the errors shown in Table 2. The QQ-plots corresponding to Pareto–Mathai distribution for the sectors with more quantity of registered events, that is, sectors 1 and 2, are compared with QQ-plots for log-gamma distribution in Figure 7. The QQ-plots showed a better fit for the Pareto–Mathai distribution than for log-gamma distribution, especially in the tails. The Pareto–Mathai distribution describes adequately the seismic moments, which is a variable typically heavy tailed [12]. As can be observed in Figure 4 and Table 2 the fits for Sectors 3 and 4 are acceptable but the proposed model exhibits important deviations from empirical data in these sectors, which may be due bimodal behavior [21].

## 5. Discussion and Conclusions

Understanding the dynamics that govern the occurrence of earthquakes, both in their generation and in their effects on cities, is a major challenge for scientists. The search for predictive models has been focused on finding the one that best fits the reliable data recorded by seismometers, i.e., earthquakes whose magnitude is over the completeness magnitude $M_{c}$. However, it is useful to model the earthquakes for the entire range of seismic moments in order to determine analytically the magnitude $M_{c}$. For these events, the Gutenberg Richter law does not fit very well and an ad hoc distribution is introduced to model it. Indeed, the introduction of Mathai’s pathway model adjusts very well to the magnitude data recorded in the lower-valued region by incorporating the density of states. But considering that the seismic moment is preferable to any magnitude scale due to its accuracy, we use the seismic moments instead of magnitudes. To do so we consider recent models that have been proposed from the point of view of statistical mechanics: non-extensive statistical mechanics (Tsallis’ model), superstatistics (Beck-Cohen model) and more recently the Mathai pathway model. (the latter has been applied with incresing frequency).

In this article, the Pareto–Mathai model was constructed by applying a transformation to the Mathai distribution. The latter is the transformation used to pass from the exponential distribution to the Pareto model. When analyzing some of its properties, the probability density was obtained first. It demonstrated that this probability density increases from an initial minimum value to an absolute maximum value and then decreases as a function of the increasing seismic moments. It was shown that, in the range of values of the entropic index q>1 (where the Pareto–Mathai distribution corresponds to a heavy-tailed distribution) there is no moment, in contrast to the Mathai distribution for which the *j*-th moment exists under the proper conditions. Additionally, we have proposed the multi-varied Pareto–Mathai distribution, as it is well-suited for complex systems. The latter are multi-parameter systems and their components could be spatially or temporally interdependent or strongly correlated to a high degree.

To apply this new model, we fit it to registered microseisms in a Chilean underground mine of a full catalog including reliable and unreliable data. Analyzing cumulative frequency, we have demonstrated that the constructed Pareto–Mathai model adjusts very well to the recorded data of microseisms that occurred in a four-year period. The good fit of the Pareto–Mathai distribution to the empirical seismic data allows us to analytically calculate the completeness magnitude, which is fundamental in order to identify authentic data and faulty measurements.

The occurrence of microseisms in underground mines has a special characteristic in terms of impact on productivity, as well as in seismology laboratories [22,23]. Thus, the understanding of this phenomenon is crucial for saving lives of mining workers, avoiding financial losses, etc. According to the scale of values of seismic magnitudes, in this type of laboratory the studies of the complex non-linear dynamics of large-scale natural events could be significantly improved.

## Figures and Tables

**Table 1:** Extreme values for each sector registered in the period 2003–2007.

	Minimum		Maximum	
Sector	Magnitude	Seismic Moment	Magnitude	Seismic Moment
1	−2.12	$8.273\times 10^{5}$	2.97	$3.553\times 10^{13}$
2	−1.52	$6.516\times 10^{6}$	2.29	$3.409\times 10^{12}$
3	−1.31	$1.375\times 10^{7}$	0.97	$3.621\times 10^{10}$
4	−1.31	$1.384\times 10^{7}$	1.68	$4.116\times 10^{11}$
5	−1.55	$5.931\times 10^{6}$	1.91	$9.190\times 10^{11}$

**Table 2:** RMSE and $R^{2}$ errors for the Pareto–Mathai distribution fit.

Sector	RMSE	$R^{2}$	MAPE(%)
1	0.00739	0.99926	2.70572
2	0.00844	0.99737	6.45977
3	0.02119	0.99415	10.12413
4	0.04437	0.99662	13.57314
5	0.01346	0.99775	3.96032
1–5	0.01519	0.99720	5.15808

**Table 3:** The magnitudes and seismic moments for which the theoretical density function is maximum.

Sector	Completeness Magnitude	Completeness Seismic Moment
1	−1.22	$1.849\times 10^{7}$
2	−0.68	$1.218\times 10^{8}$
3	−0.99	$4.062\times 10^{7}$
4	−0.77	$8.744\times 10^{7}$
5	−1.08	$3.007\times 10^{7}$

## Data Availability

The data is confidential but can be provided if it is requested.
